# Combining Polymer and Cyclodextrin Strategy for Drug Release of Sulfadiazine from Electrospun Fibers

**DOI:** 10.3390/pharmaceutics15071890

**Published:** 2023-07-05

**Authors:** Diego C. Morais, Marina L. Fontes, Analú B. Oliveira, Paulo R. Gabbai-Armelin, Túlio M. Ferrisse, Luiz F. C. De Oliveira, Fernanda Lourenção Brighenti, Hernane S. Barud, Frederico B. De Sousa

**Affiliations:** 1 Laboratório de Sistemas Poliméricos e Supramoleculares (LSPS), Instituto de Física e Química, Universidade Federal de Itajubá (UNIFEI), Itajubá 37500-903, MG, Brazil; diegocapronimorais@gmail.com; 2 Laboratório de Biopolímeros e Biomateriais, Universidade de Araraquara (UNIARA), Araraquara 14801-340, SP, Brazil; marinafontes29@gmail.com (M.L.F.); hernane.barud@gmail.com (H.S.B.); 3 Department of Morphology and Pediatric Dentistry, School of Dentistry, São Paulo State University (UNESP), Araraquara 14801-903, SP, Brazil; analu.oliveira@unesp.br (A.B.O.); paulogabbai@gmail.com (P.R.G.-A.); f.brighenti@unesp.br (F.L.B.); 4 Department of Dental Materials and Prosthodontics, School of Dentistry, São Paulo State University (UNESP), Araraquara 14801-903, SP, Brazil; tuliomferrisse@gmail.com; 5 Núcleo de Espectroscopia E Estrutura Molecular—Departamento de Química—ICE, Universidade Federal de Juiz de Fora (UFJF), Juiz de Fora 36036-900, MG, Brazil

**Keywords:** electrospinning, nanomaterials, supramolecular systems, drug delivery, in vitro

## Abstract

This study reports the fabrication of polymeric matrices through electrospinning using polymethyl methacrylate (PMMA) and poly(lactic-co-glycolic acid) (PLGA), biocompatible polymers commonly used in medical systems. These polymers were combined with an antibacterial drug, sulfadiazine sodium salt (SDS) or its supramolecular system formed with hydroxypropyl-β-cyclodextrin (HPβ/CD) at 1:1 molar ratio, aiming to assemble a transdermal drug delivery system. The formation of fibers was confirmed by scanning electron microscopy (SEM), and the fibers’ surface properties were analyzed using contact angle and water vapor permeability techniques. Drug release tests and cell viability assays were performed to evaluate the potential toxicity of the material. SEM images demonstrated that the obtained fibers had nanoscale- and micrometer-scale diameters in PLGA and PMMA systems, respectively. The contact angle analyses indicated that, even in the presence of hydrophilic molecules (SDS and HPβCD), PMMA fibers exhibited hydrophobic characteristics, while PLGA fibers exhibited hydrophilic surface properties. These data were also confirmed by water vapor permeability analysis. The drug release profiles demonstrated a greater release of SDS in the PLGA system. Moreover, the presence of HPβCD improved the drug release in both polymeric systems and the cell viability in the PMMA SDS/HPβCD system. In terms of antibacterial activity, all membranes yielded positive outcomes; nevertheless, the PLGA SDS/HPβCD membrane exhibited the most remarkable results, with the lowest microbial load values. Additionally, the pseudo wound healing analysis demonstrated that the PLGA SDS/HPβCD fiber exhibited results similar to the control group. Consequently, these findings exemplify the substantial potential of the obtained materials for use in wound healing applications.

## 1. Introduction

Skin wounds are lesions that can be caused by a variety of factors, including cuts, scrapes, punctures, diabetes, or burns, which can be associated with trauma, infections, autoimmune diseases, and so on [1,2]. However, burns and diabetes can be considered the most common sources of infectious skin lesions, with diabetes actually worsening the injury and affecting wound healing due to blood circulation issues [3]. On the other hand, skin burns are injuries caused by heat, electricity, radiation, chemicals, or radioactive substances, and their severity depends on the intensity of the heat source, the affected skin area, and the depth of the injury [4]. Therefore, wound healing is a complex process that the body undergoes to repair damaged tissue.

The treatment for a wound depends on several factors, including the cause, size, location, and type of wound, typically involving wound cleaning and covering it with dressing material to promote healing [2,5]. Among various treatment options, those that use membrane-containing healing substances, such as proteins, vitamin C, hyaluronic acid, and anti-inflammatory agents, such as topical corticosteroids, are becoming increasingly popular. These dressings serve to protect wounds from external influences and are considered essential for healing and skin recovery [6]. When applied, these healing agents increase the efficacy of the dressing by improving the control of microorganisms in the wound area. This is achieved by reducing the proliferation of bacteria and/or fungi as microbial presence can slow down the healing process [7]. Among the various categories of compounds applied to inhibit the growth of microorganisms in skin lesions and to act as bactericidal and bacteriostatic agents, the classes of beta-lactams, aminoglycosides, macrolides, and sulfonamides stand out [8,9].

**Figure 1:**
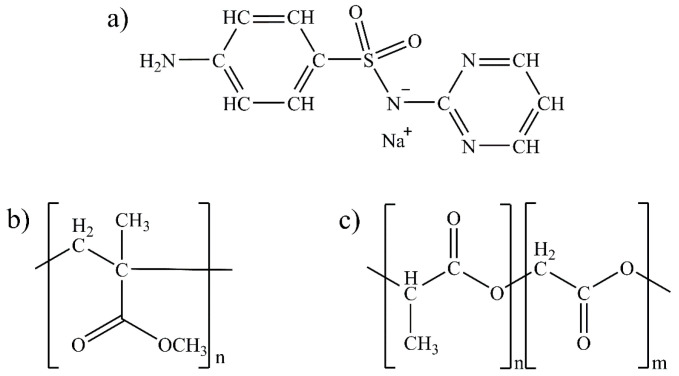
Chemical structures of (**a**) SDS, (**b**) PMMA monomer, and (**c**) PLGA monomer.

Sulfonamides are one of the main therapeutic groups used in the treatment of bacterial infections [10]. The -SO_2_NH group in their structure is frequently explored in studies for the development of new drugs as it has a toxophoric function that confers antimicrobial activity to sulfonamides [11]. Among sulfonamides, sulfadiazine sodium salt (SDS) (Figure 1a) stands out for its action of inhibiting the production of folic acid inside bacterial cells. Additionally, SDS has infection control properties that aid in the wound healing process by inhibiting bacterial proliferation, reducing infections, and thereby increasing the replication of keratinocytes [12]. Then, in order to promote the drug delivery of sulfonamides, biocompatible and biodegradable polymers have been used.

Among them, poly (methyl methacrylate) (PMMA, Figure 1b) is a treatment used to promote wound healing and reduce scar formation. It is applied directly to the affected area, forming a protective barrier that prevents infections and reduces inflammation. PMMA can also help absorb excess fluids and exudates from the wound, which may help accelerate the healing process [13]. Several applications have been found in the medical field for this polymer, including tissue reconstructions [14], drug delivery systems [15], and even the production of dental prostheses [16]. These applications are possible due to their biocompatibility, low toxicity, and high mechanical strength [17]. Beyond PMMA, poly (lactide-co-glicolide) (PLGA, Figure 1c), which is obtained by copolymerizing lactic acid and glycolic acid, is characterized by its high biocompatibility and easy degradation in the environment depending on the ratio of acid and glycolic monomers, which generate lactate and glycolate salts as final products. For this reason, PLGA is approved by the Food and Drug Administration and widely used in delicate procedures in the healthcare field, such as labeling cancer cells and drug delivery [18].

An interesting strategy to improve polymer uses in drug delivery systems is related to their blend with natural or chemically modified cyclodextrins (CDs), such as hydroxypropyl-β-cyclodextrin (HPβCD) [19]. This system can enhance the interaction between the drug molecule and matrix due to its inclusion into the hydrophobic cavity of CD, allowing for the modulation of drug release [20]. In order to combine supramolecular systems and polymer devices, electrospinning has been successfully used. Electrospinning is an efficient method for producing fibers with diameters ranging from nanometers to micrometers [21]. These fibers, obtained from natural or synthetic polymers, have several desirable properties that make them suitable for biomedical applications [22,23]. Moreover, these fibers present a high surface area to volume ratio, can be porous, and mechanically resistant [24,25]. These properties allow these fibers to be used as slow drug release systems, reducing dosage errors, thereby increasing efficacy and reducing toxicity and side effects [26]. Herein, PMMA and PLGA polymers were electrospun into fibers in the presence of SDS (PMMA SDS and PLGA SDS) and with the supramolecular complex of this drug molecule with HPβCD (PMMA SDS/HPβCD and PLGA SDS/HPβCD). These polymeric materials were characterized in terms of morphology and surface properties and tested in vitro in order to evaluate their drug release profile, cell cytotoxicity, and wound healing. In vitro wound healing (also called scratch assay) is usually used in order to quantify the cell migration on a two-dimensional surface over time, and in this case, for all electrospun materials. In this sense, images over time were used to identify the closure of the scratch area due to cell migration. Moreover, the materials’ bioactivity and antibacterial activity were also screened for *Staphylococcus aureus* (*S. aureus*) and *Escherichia coli* (*E. coli*)

## 2. Materials and Methods

### 2.1. Reagents

Sulfadiazine sodium salt (SDS) was purchased from the Sigma-Aldrich (São Paulo, Brazil); poly(metyl metacrylate)—100 kDa was purchased from the Polysciences (Warrington, PA, USA); poly (D,L lactide-co-glicolide) (50:50)—60 kDa, was purchased from the Lactel (Birmingham, AL, USA); hydroxypropyl-β-cyclodextrin (substitution degree 5–8 and Mw ≈ 1400 g/mol) was obtained from Cerestar Company (Decatur, AL, USA); N,N-dimethylformamide (DMF), was purchased from the Êxodo Científica (Sumaré, Brazil); tetrahydrofuran (THF) was purchased from the Vetec (Duque de Caxias, Brazil); and chloroform (CHCl_3_) was purchased from the Synth (Rio de Janeiro, Brazil).

### 2.2. Fabrication of Fibers by Electrospinning

Polymer solutions were prepared as follows: 1.8 g of PMMA (using DMF/THF in a 70:30 ratio) or PLGA (using DMF/CHCl_3_ in a 1:1 ratio); both solutions were at 30% *w*/*v*. The electrospun polymer solutions containing SDS and SDS/HPβCD were prepared under the same conditions. The amount of SDS used was 10.0 mg and for the supramolecular complex system, the 1:1 molar ratio was considered (10.0 mg of SDS and 52.0 mg of HPβCD). Table 1 depicts the electrospun parameters applied to obtain the polymeric fibers.

### 2.3. Contact Angle Goniometry

The surface properties of the fibers were investigated on the upper surface of the electrospun mats using a contact angle measuring system (Kruss G10, Heidelberg Germany) and the sessile drop method, at room temperature, using 10 × 10 μL droplets of ultrapure water (Milli-Q^®^ water, São Paulo, Brazil) for each fiber sample. The contact angle values presented in the discussion section were obtained by the arithmetic mean calculations, and the standard deviation of the 10 angles was obtained from the analysis.

### 2.4. Scanning Electron Microscopy

The electrospun fibers morphology study was carried out by scanning electron microscopy (SEM) FEI TECNAI G2 and Quanta 200 FEG-FEI operated at 5 kV. Dried samples were gold coated with 5 nm using BAL-TC MC5010 automated sputter coater. The diameters of all fibers were measured from SEM images, using at least 60 measurements from each of three different micrographs, by ImageJ 1.52a version analysis software.

### 2.5. Water Vapor Permeability

The water vapor permeability (WVP) measurements for all fibers were performed according to the literature [27]. Fiber samples were cut into circular shapes, approximately 1.0 cm in diameter, and placed in glass tube caps, which were filled with 10.0 mL of Milli-Q^®^ water. Fiber thicknesses were evaluated using a digital micrometer (±0.001 nm) in triplicate, and the average and standard deviation of these values were used. Each tube containing water and the fibers on the top of the glass was weighed and placed in a vacuum oven at 25 °C. The weight loss for each system was monitored during 0, 24, 48, 72, 96, 120, and 168 h. This test was performed in duplicate, and the mean values were calculated. The water vapor flow (J) was calculated using Equation (1):

(1) $$J=\frac{∆m}{∆t\times A}$$

where, Δm/Δt represents the angular coefficient of mass variation over time, which is obtained through the graph generated from the results of gravimetric measurements of the samples. (A) is the sample surface area (in cm^2^) that is exposed during the test. The WVP was calculated using Equation (2):

(2) $$WVP=\frac{J\times L}{∆P\times (T)}$$

where J was obtained from Equation (1), L is the membrane thickness, and ΔP × (T) is the vapor pressure difference generated inside the oven at 25 °C.

### 2.6. Drug Content and In Vitro Drug Release

Prior to the drug release study, drug load capacity was determined by an extraction method for both groups (PMMA and PLGA fibers containing SDS). In this sense, 50 mg of each fiber (PMMA SDS, PMMA SDS/HPβCD, PLGA SDS, and PLGA SDS/HPβCD) was dissolved in 4.0 mL of CHCl_3_ and 4.0 mL of Milli-Q^®^ water. The solution was stirred and, after 24 h, SDS was quantified from the aqueous phase using a UV-vis spectrophotometer. The UV-vis spectra were obtained from 200 to 800 nm, and the SDS concentration was analyzed at 260 nm.

For the release study, a modified Franz Cell [28] assembly was used, which is a diffusion system with temperature control through a flow of water in a thermostatic bath. The system consists of glass cells with a donor compartment where the sample is placed, a receiver compartment, which is filled with a phosphate buffer saline (PBS) solution (pH 7.4), and a dialysis membrane (3 cm^2^ diameter acetate membrane), which is inserted between these compartments to allow the flow between the sample and the receptor solution. The Franz Cell system was incubated and maintained under agitation and a constant temperature of 37 °C. The sample remained in contact with the solution for a period of 24 h, and at predetermined times (0, 7, 15, 30, 60, 120, 240, 480, and 1440 min), aliquots of 0.8 mL were collected, and the same volume of pure receptor solution was replaced in each of the cells. The aliquots were analyzed by UV-Vis at 260 nm to determine the SDS release from each electrospun fiber material. All experiments were performed in six replicates, and graphs were obtained that demonstrate the release profile.

### 2.7. Cell Cytotoxicity

Initially, fibroblast cells (L929) were cultured in Dulbecco’s Modified Eagle’s Medium (DMEM) (Embriolife, São Paulo, Brazil) supplemented with 10% Fetal Bovine Serum (FBS) and antibiotics (penicillin 100 U/mL; streptomycin 0.1 mg/mL), followed by incubation at 37 °C and 5% CO_2_. L929 (NCTC clone 929) was purchased from ATCC and generously provided by the Monoclonal Antibody Laboratory (Sao Paulo State University, UNESP, Araraquara, SP, Brazil). After two consecutive passages, the cytotoxicity assay was initiated using a concentration of 1.5 × 10^4^ cells/well, which were incubated in a 96-well plate. The plate was kept in the incubator for 24 h, maintaining the same temperature and CO_2_ percentage as described above.

Simultaneously to the cell plating, the extraction media (treatments) using the polymeric fibers of PLGA and PMMA with or without SDS or SDS/HPβCD were prepared according to the literature [29]. For this purpose, 6 cm^2^/mL of each material was placed in a 1.5 mL microtube with the addition of 1.0 mL of culture medium (DMEM + 10% fetal bovine serum). The materials were kept under agitation for 24 h at 37 °C. After the agitation period, the media were filtered through a 0.22 µm syringe filter. After filtering, the extraction media (0.1 mL per well) were placed in contact with the cell monolayer, followed by incubation in a CO_2_ incubator for 24 h. After the 24 h treatment period, the extraction media were removed from the wells, followed by two consecutive washes with PBS. Then, 50.0 μL of MTT (3-(4,5-dimethylthiazol-2-yl)-2,5-diphenyltetrazolium bromide) previously diluted to a concentration of 1.0 mg/mL was added to each well. The plates were incubated at 37 °C, and protected from light, until the observation of violet formazan crystals formation (3 h). After the incubation period, the MTT was removed from the wells, and the formazan crystals formed were dissolved by adding 100 μL of absolute isopropyl alcohol.

Absorbance was obtained using a microplate spectrophotometer, with a wavelength of 570 nm. As a positive control for cell death, the cells were treated with 10% DMSO (dimethyl sulfoxide), and as a negative control for survival, the cells were treated with DMEM + 10% FBS. The experiments were conducted in triplicate for each sample in three independent assays, including the controls [30]. Absorbance values were used to determine the mean percentage of cell viability relative to the survival control, as presented in Equation (3) [29].

(3) $$\%\;cell\;viability=\frac{\left(Abs\;extraction\;media-Abs\;blank\right)}{\left(Abs\;positive\;control-Abs\;blank\right)}\times 100$$

### 2.8. In Vitro Wound Healing

Fibroblast cells (L929) were cultured following the same procedure described in Section 2.7. While the cells were plated, extraction media (treatments) were prepared using PLGA and PMMA polymeric fibers, with or without SDS or SDS/HPβCD, following the standard [31]. For this, 6 cm^2^/mL of each material was placed in a 1.5 mL microtube, and 1.0 mL of culture medium (10% FBS) was added. The materials were agitated for 24 h at 37 °C, and then media were filtered through a 0.22 µm syringe filter.

After plating, the cell monolayer had its central region removed by a scratch using a pipette tip. After scratching the central region, the culture medium was removed, and the wells were washed twice with PBS. Then, the filtered extraction media were placed in contact with the monolayer, and images were recorded. The cells were kept under incubation, and micrographs were recorded at predetermined periods of 2, 4, 6, 8, 10, and 24 h [32]. The obtained images were analyzed, and the area of the initial scratch, as well as at the other determined times, was determined using ImageJ^®^ software (1.52a version). The closure (%) of the scratch was calculated according to Equation (4):

(4) $$\%\;wound\;closure=\frac{\left[Area\;t\left(0\right)-Area\;t\left(x\right)\right]}{Area\;t(0)}\times 100$$

### 2.9. Primary Screening for Material Bioactivity

The initial screening of materials was conducted using the agar diffusion technique following the guidelines of CLSI M2-A8 [33], with the exception that antibiotic discs were replaced with the materials themselves or GR1 qualitative filter paper discs (Whatman). The following groups were evaluated: negative control—filter paper disc soaked in culture medium (C); chlorhexidine—filter paper disc soaked in 0.12% chlorhexidine digluconate solution (CHX); group 1—PMMA; group 2—PLGA; group 3—PMMA SDS; group 4—PMMA SDS/HPβCD; group 5—PLGA SDS; group 6—PLGA SDS/HPβCD. A circular cutter (Ø 16 mm) was used to prepare samples, ensuring consistent diameter and thickness across the different study groups.

The following reference strains were used: *Staphylococcus aureus* (*S. aureus*) ATCC 25923 and *Escherichia coli* (*E. coli*) ATCC 25922. The strains were obtained from the Oswaldo Cruz Foundation (FIOCRUZ) and were maintained in soybean tryptone broth (TSB) at −80 °C. Reactivation of the strains was carried out using TSB agar under aerobic conditions at 37 °C. An inoculum of each studied microorganism was prepared by adjusting the optical density reading at 630 nm to 0.08–0.10 (approximately 1 × 10^8^ cells/mL).

The prepared inoculum was evenly spread on the surface of TSB agar and allowed to rest at room temperature for 3 min. Subsequently, the previously labeled samples were placed equidistantly on the agar, and after a 15 min interval, the plates were incubated in a bacteriological incubator at 37 °C for 18 h. The experiments were conducted on two separate occasions (n = 4/group).

### 2.10. Evaluation of Antibacterial Activity in Biofilms

After the initial screening, single-species biofilms of *E. coli* and *S. aureus* were developed on the bottom of pre-prepared 24-well plates using the previously described growth conditions [34]. A volume of 150 µL of bacterial suspensions, prepared as described in Section 2.9, was added to each well of the plates. Simultaneously, specimens from each experimental group (n = 4/group) were individually placed in the wells of the 24-well plate during biofilm formation. The plates were incubated for 24 h at 37 °C with orbital agitation (75 rpm). After 24 h of incubation, samples from each experimental group were removed from the wells containing biofilms and analyzed using standard biochemical and microbiological analysis methods. These methods were used to determine the dry weight of the biofilm (biomass) and colony-forming units (CFU).

The biofilms adhered to the samples were dispersed in 2 mL of 0.89% NaCl. An ultrasonic bath (42 kHz) was used for 10 min in glass tubes. Then, the dispersed biofilms were transferred to new tubes. To ensure the complete transfer of dispersed microorganisms, the glass tubes were rinsed with 3 mL of 0.89% NaCl, which was also added to the dispersed biofilms, resulting in a total volume of 5 mL of the biofilm suspension. The suspension was homogenized for 30 s at 7 watts (Q125 Sonicator model, QSonica) and processed. From the 5 mL volume, 0.1 mL was used for serial dilution and plating on TSA agar, followed by incubation for 24 h at 37 °C for CFU counting.

For dry weight determination, the remaining volume (4.9 mL) was centrifuged at 4000 rpm for 20 min (4 °C). The supernatant was discarded, and the pellet was washed with 2 mL of MilliQ^®^ water twice, under the same conditions as described (4000 rpm/20 min/4 °C). After the wash, the pellet was resuspended in 1 mL of water, transferred to pre-weighed aluminum foil containers, and dried at 100 °C for at least 4 h. After drying, the containers were weighed again. The insoluble dry weight was calculated by taking the difference in mass before and after weighing and multiplying it by the sample volume divided by the total volume.

### 2.11. Statistical Analysis

One-way was used to determine the statistical significance, and the data and images were processed using GraphPad Prism 8 software. For microbiological analyses, a descriptive analysis of the data was performed. Since the sample size = 4, the Kruskal-Wallis test followed by Dunn’s multiple comparison test was used. All statistical analyses were performed at α = 0.05 level of significance.

## 3. Results and Discussion

### 3.1. Morphological Analyses

**Figure 2:**
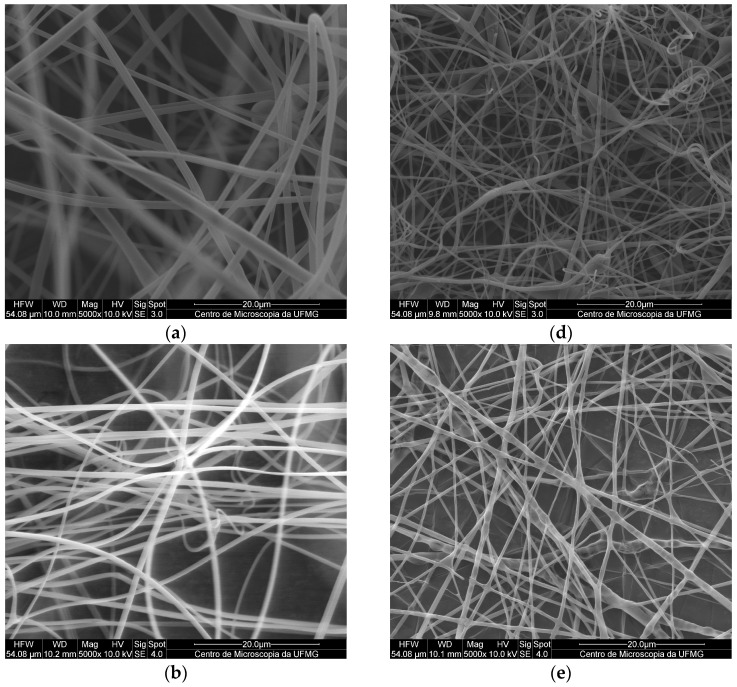

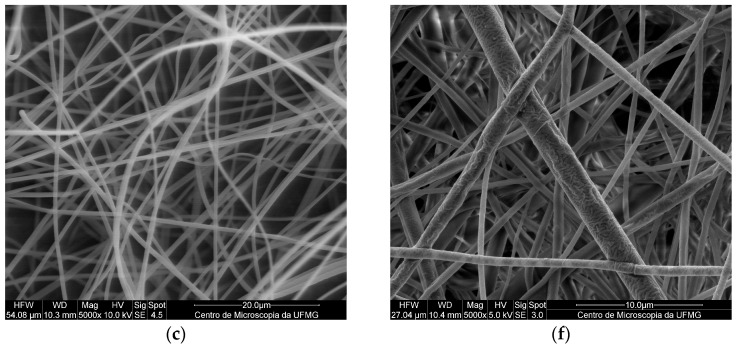
**SEM** images of fibers obtained by electrospinning with a magnification of 5000×: (**a**) PMMA, (**b**) PMMA SDS, (**c**) PMMA SDS/HPβCD, (**d**) PLGA, (**e**) PLGA SDS, (**f**) PLGA SDS/HPβCD.

The micrographs of the electrospun PMMA fibers (Figure 2a–c) indicated the absence of pores, holes, and spindles, as well as a lack of droplets or particles. The pure PMMA fibers presented an average diameter of 1.55 ± 0.13 µm, which was in agreement with previously reported results [15]. Comparing this result with those obtained for the PMMA SDS and PMAA SDS/HPβCD fibers, no significant difference in average diameter was observed (1.40 ± 0.22 µm for PMAA SDS and 1.35 ± 0.26 µm for PMMA SDS/HPβCD). These data suggest that the concentration of SDS used or its supramolecular complex with HPβCD did not affect the electrospinning process of the PMMA polymer.

Like the PMMA fibers, the electrospun materials using PLGA (Figure 2d–f) did not present pores or holes; however, small amounts of spindles could be found in some areas. The pure PLGA fibers presented an average diameter distribution of 1.15 ± 0.28 µm, while a significant change in the average diameter was observed for the PLGA SDS fibers (0.81 ± 0.31 µm). The PLGA SDS/HPβCD fibers showed a reduction in the average diameter, a twofold reduction compared with that observed for the PLGA SDS fibers (0.48 ± 0.20 µm). There is a variation in the mean values of diameter distribution, demonstrating significant differences when comparing the pure PLGA fibers with those containing SDS or SDS/HPβCD. According to Schoeller et al. [35], the addition of salt molecules into the PLGA polymer solutions can increase the conductivity of this solution, consequently, reducing the diameters of the fibers from a micrometric to a nanometric scale.

In addition to the electrospun morphologies, histograms of each polymer system, PMMA (Figure S1) and PLGA (Figure S2), were plotted. PMMA electrospun fibers presented a maximum size distribution value of approximately 1.5 µm, while the PMMA SDS and PMMA SDS/HPβCD electrospun fibers showed a size distribution varying from 0.8 to 1.8 µm, suggesting that the drug and the supramolecular complex can affect the polymer solution characteristics. On the other hand, the presence of the supramolecular system (SDS/HPβCD) made the average distribution of diameters smaller for this polymeric system than the PLGA pure fibers (from 0.4 µm to 1.0 µm).

### 3.2. Surface Properties

In order to investigate the surface properties of PMMA and PLGA fibers, the water contact angle analysis was employed [36]. This technique allows the determination of the surface characteristics of electrospun materials, indicating whether these have hydrophilic or hydrophobic properties. The contact angle images of the water droplets on the surface of all electrospun materials using PMMA are depicted in Figure S3.

The contact angle value (θ) obtained for pure PMMA fibers is 136.2 ± 2.1°, a value that is similar to the PMMA SDS and PMMA SDS/HPβCD fibers, 140.1 ± 1.3° and 139.6 ± 2.3°, respectively, indicating that the fibers have hydrophilic surface characteristics. These results are in agreement with the distribution of average diameters for electrospun PMMA systems, in which no significant difference is found in the average diameter values for these materials. Conversely, all PLGA polymer fiber systems have presented hydrophilic surface characteristics. Figure S3 depicts the video frame images captured from pure PLGA electrospun fibers, in which after 1.7 s most of the water droplets were already adsorbed by the material. The hydrophilic surface characteristics of PLGA fibers have been reported previously [37], and the present material was increased based on the average diameter of the electrospun fibers and the existence of hydrophilic molecules on the surface of the fibers.

In addition to the contact angle analyses, WVP analysis was also carried out, which involved the measurement of the rate of water vapor transmission through a membrane. These values can be used to understand drug diffusion models in polymeric matrices, analyze interactions between system components, and even predict drug behavior within the matrix [38]. According to Bertuzzi [38], permeability is a property that results from the diffusivity and solubility of a system, and its behavior follows Fick or Henry’s laws. The interaction between water vapor and the polymeric matrix can affect the solubility or diffusion of the water molecule through the matrix. A variety of factors can influence permeability, including the hydrophobic or hydrophilic nature of the material, structural modification of the material, and the presence of pores or fissures in the membrane [39]. Table 2 presents the WVP values for both electrospun polymeric systems (PMMA and PLGA), as well as their associations with the SDS and the SDS/HPβCD supramolecular system, along with the average membrane thickness and their respective standard deviations.

Comparing the PMMA fiber groups, it was observed that there was no significant difference between the PMMA and PMMA SDS fibers; however, the PMMA SDS/HPβCD system showed a significant difference. The increased value observed for the supramolecular system can be correlated to the presence of a highly soluble complex on the surface of the fibers, compared with the other PMMA systems. The PLGA systems were characterized as hydrophilic based on the water contact angle analysis with the material surface, which was also verified according to the WVP. The PLGA SDS/HPβCD presented the highest WVP value among all materials, which was attributed to the presence of the supramolecular complex. The presence of these highly water-soluble molecules in the polymeric fibers was more significant for the observed WVP values than for those related to the average fiber diameter [10].

### 3.3. Surface Properties

The in vitro release assay for fibers containing SDS was conducted to assess the possibility of these systems acting as controlled release systems. First of all, SDS was quantified in all systems. In this sense, two calibration curves were obtained using a UV-vis spectrophotometer for SDS in an aqueous solution, with and without the presence of HPβCD (Figure S4). The experiments were performed in triplicate, and the maximum absorbance value at 260 nm was used to construct the calibration curves. The data shown in Figure S4 represent the average of different solutions, and the standard deviation is calculated from these data. Table 3 presents the drug loading percentage for each system, obtained by the UV-vis analysis.

**Figure 3:**
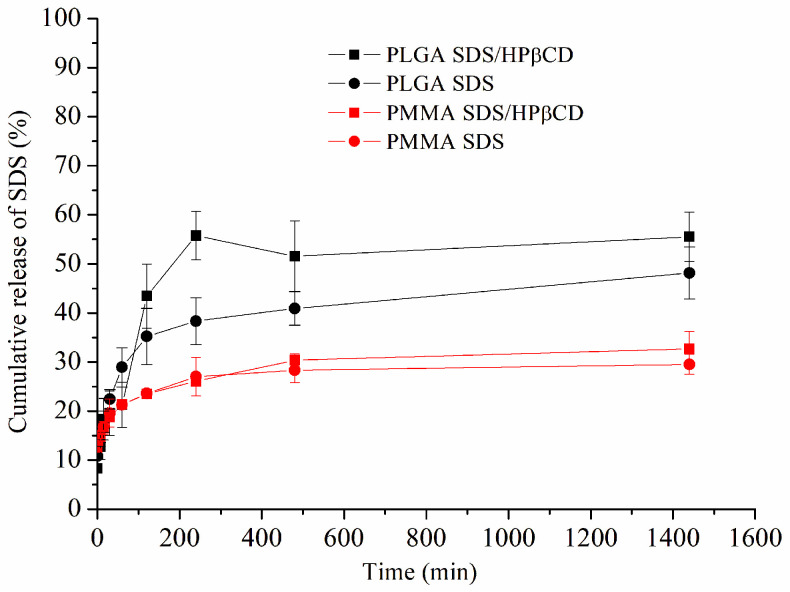
Cumulative in vitro drug release profiles of the PLGA SDS, PLGA SDS/HPβCD, PMMA SDS, and PMMA SDS/HPβCD fibers.

The drug release profile for each PMMA and PLGA system using the Franz Cell release system is depicted in Figure 3. Comparing both polymers (PMMA and PLGA), it is possible to observe a small amount of cumulative SDS release from both PMMA SDS and PMMA SDS/HPβCD fibers. Moreover, no significant difference was observed when comparing both PMMA polymeric systems, in which the maximum SDS cumulative release was limited to 30%. These results suggest that the presence of HPβCD was not capable of affecting or improving the SDS release using PMMA. However, the PLGA SDS/HPβCD presented the highest cumulative drug release, in which the maximum drug release was about 56%, while the PLGA SDS fibers demonstrated a maximum drug release of 38% after 240 min. These differences in the cumulative drug release when comparing the PLGA fiber systems can be correlated to the observed fibers’ size distribution, which varied from 0.81 ± 0.31 to 0.48 ± 0.20 µm for the PLGA SDS and PLGA SDS/HPβCD, respectively. In addition, the higher WVP values verified for the PLGA SDS/HPβCD fibers can affect the cumulative drug release, and the presence of HPβCD can improve the SDS release [40].

In order to evaluate the SDS release mechanism, experimental data were fitted into various kinetic models: First Order, Higuchi, Peppas, Weibull, Zero Order, and Hixon and Crowell (Table 4). These results, for all polymeric fibers, were best fitted to the Peppas model. The n values were calculated for all electrospun materials (n < 0.5). This result indicated a Pseudo-Fickian diffusion mechanism. It was found that the presence of HPβCD resulted in an increase in the k values of the fibers, supporting the reduction in diameter and greater permeability, considering that a larger surface area could enhance drug release, which would consequently increase the diffusion coefficient [41,42].

### 3.4. Cell Cytotoxicity

**Figure 4:**
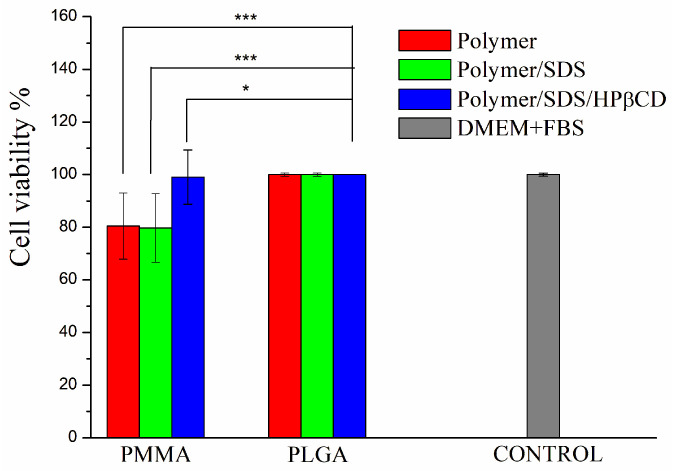
Percentage of cell viability of L929 cells for PMMA and PLGA fiber systems. The results are presented as mean ± standard deviation. * *p* < 0.05, *** *p* < 0.001.

The cell viability assay for all fibers aimed to evaluate whether polymeric membranes exhibited cellular toxicity. It has already been reported that PMMA and PLGA polymers do not exhibit cellular toxicity and are considered biocompatible [15,43]. In order to determine cell viability, the MTT assay was carried out, which consisted of evaluating the metabolic activity of cells. As presented in Figure 4, it can be observed that the materials did not exhibit cytotoxicity. Comparing the PMMA systems, comparable cell availability of PMMA SDS/HPβCD to the control can be observed. A greater variation is observed for PMMA fiber systems, and lower cell viability has been reported for polymeric systems obtained using PMMA, including those systems obtained by electrospinning [44,45]. Moreover, no statistical difference is found for all PLGA systems. According to ISO 10993-5 [29], cell viability must be higher than 70% for tested materials/medical devices to be considered nontoxic. As reported in the literature, PLGA has already been used in formulations that show improved cell viability and is being considered a gold standard polymer for drug release [46].

### 3.5. In Vitro Wound Healing

**Figure 5:**
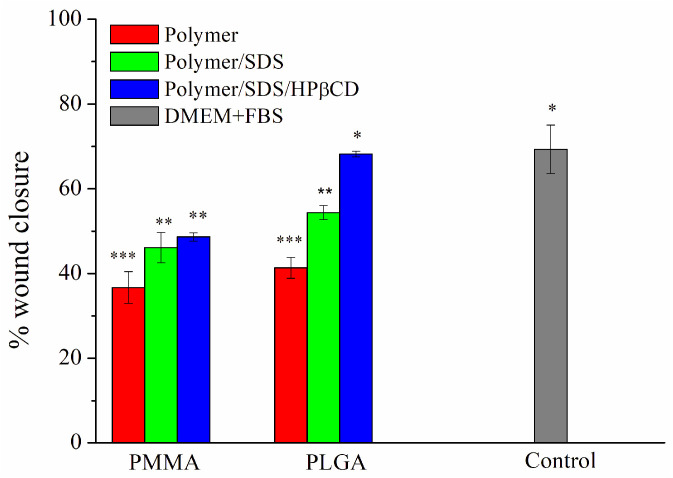
Percentage of pseudo-wound closure of L929 cells for PMMA and PLGA fibers systems. The results are presented as mean ± standard deviation. * *p* < 0.05, ** *p* < 0.01, *** *p* < 0.001.

The cell migration percentage was investigated to quantify the gap closure. Figure 5 depicts the percentage of wound closure after 24 h using all electrospun materials. It was observed that the presence of the SDS drug accelerated cell migration in both polymer systems (PMMA and PLGA) compared with pure electrospun fibers. Furthermore, when compared with systems containing the supramolecular system, cell migration was higher for the PLGA SDS/HPβCD, comparable to the control group (about 68%). This result supports the higher permeability values of these systems and, consequently, the greater drug release observed. Figures S5 and S6 present the optical microscopy images for cell migration using all studied polymeric systems at 0, 10, and 24 h.

### 3.6. Primary Screening for Material Bioactivity

Among the materials examined, only one (PLGA SDS/HPβCD) exhibited an inhibition halo that was equal to or larger than the positive control (CHX) for both *E. coli* and *S. aureus* (Table 5). It is worth noting that the inhibition halo method is considered semi-quantitative, and the size of the halo does not directly correlate with antimicrobial activity [47]. Taking into account the unique characteristics of the materials under evaluation, we opted to assess all groups based on their inhibition of biofilm formation.

### 3.7. Evaluation of Antibacterial Activity in Biofilms

**Figure 6:**
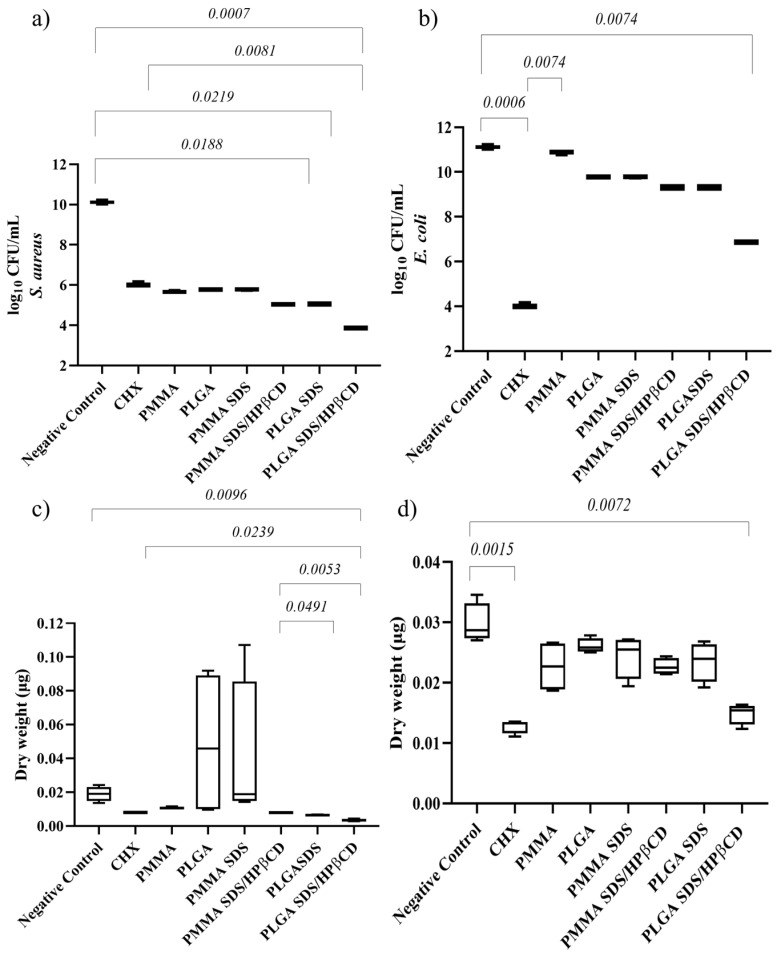
Median plot with minimum/maximum values illustrating the significant results of Dunn’s multiple comparison test for log CFU/mL of (**a**) *S. aureus* and (**b**) *E. coli* biofilms. Median plot with minimum/maximum values illustrating the significant results of Dunn’s multiple comparison test for dry weight of (**c**) *S. aureus* and (**d**) *E. coli* biofilms.

For *S. aureus* biofilms, the lowest microbial load value was observed in the PLGA SDS/HPβCD group, which was also the only group that demonstrated a statistically significant difference compared with CHX, (Figure 6a). The PLGA SDS and PLGA SDS/HPβCD groups also showed significant differences compared with the negative control group. In terms of dry weight (Figure 6c), only the PLGA SDS/HPβCD group exhibited a statistically significant difference compared with the negative control. There were no statistically significant differences between this group and the positive control (CHX).

For *E. coli* biofilms, the lowest microbial load and biomass values were found in the PLGA SDS/HPβCD group, which showed no statistically significant difference compared with CHX. Both groups demonstrated statistically significant differences when compared with the negative control group (Figure 6b). Regarding dry weight (Figure 6d), the other groups did not show significant differences compared with the control groups.

Considering the previously discussed limitations of the halo of inhibition technique, we made the decision to include all groups in the biofilm test, even though some of them exhibited a negative halo of inhibition. This approach proved to be strategic as it enabled us to identify reductions in microbial load within the groups that did not show the formation of an inhibition zone. These findings emphasize the limitations of the zone of inhibition technique, highlighting that it should only be used for initial material screening.

As depicted in Figure 6, the polymers themselves already displayed anti-biofilm activity. This can be attributed to the hydrophobic nature of the biofilms. However, there were no statistically significant differences between the polymer fibers and the control group. It is possible that these results are more related to the inhibition of bacterial adhesion than the antimicrobial activity itself, whereas the reductions in the SDS groups can be attributed to the antimicrobial activity of SDS and the inhibition of adhesion. The enhanced anti-biofilm activity observed in the PLGA SDS/HPβCD group can be attributed to the higher cumulative release of SDS compared with the other groups, as well as the higher WVP observed for this group, which may indicate the presence of soluble complexes on the fiber surface. The lower performance of the PMMA SDS group compared with the PLGA SDS/HPβCD can be explained by the lower release of SDS for the PMMA SDS system.

The comparable performance of the systems to CHX is of significant importance since we are comparing a freely available and soluble drug (CHX) with a controlled release system that achieved a maximum of 56% SDS release. This highlights the effectiveness of the methodology used to prepare the electrospun fibers. Overall, the results indicate that the developed materials, especially PLGA SDS/HPβCD, have the potential for use in the treatment of skin infections.

## 4. Conclusions

Polymeric fibers loaded with SDS were successfully obtained using the electrospinning technique. Their morphological properties were evaluated, and a decrease in the diameter of the polymeric fibers at the nanoscale level was observed in the presence of SDS and the SDS/HPβCD system using PLGA. No changes in the contact angle values were observed for both polymer systems tested based on the addition of SDS or its supramolecular complex. However, the presence of the drug and the supramolecular system directly affected the barrier properties of the polymeric membranes, increasing their WVP compared with the pure polymeric fibers. The release of SDS from the loaded fibers was mainly controlled by pseudo-Fickian diffusion, with maximum release occurring at 4 h and continuing until 24 h, demonstrating the existence of a controlled release profile. Moreover, the PLGA systems demonstrated a higher drug release, compared with the PMMA fibers. Cell viability assays were performed for all materials, and the results demonstrated that all fibers were considered nontoxic. In the wound healing test, the loaded polymeric membranes proved to be effective in promoting fibroblast proliferation, making the PLGA SDS/HPβCD polymeric fibers comparable to the control group. Finally, in the antimicrobial assays, the studied materials demonstrated effectiveness against *S. aureus* and *E. coli* bacteria. Once again, the PLGA SDS/HPβCD polymeric system showed values significantly similar to the positive control. Therefore, based on the presented results, these materials show promise for use in the wound healing process.

## Figures and Tables

**Table 1:** Parameters used for the fabrication of electrospun fibers, with and without SDS or SDS/HPβCD supramolecular systems.

Fibers	Flow Rate (mL/h)	DC Voltage (kV)	Collector Distance (cm)
PMMA	2.0	15	24
PMMA SDS	1.5	14	16
PMMA SDS/HPβCD	1.4	14	16
PLGA	1.2	19	14
PLGA SDS	1.0	15	18
PLGA SDS/HPβCD	1.0	19	12

**Table 2:** Values of water vapor permeability (WVP) and standard deviation (SD) for the electrospun fibers.

Fibers	Thickness (mm)	WVP (g mm h^−1^ m^−2^ Pa)·10^−7^
PMMA	0.262	4.29 ± 0.35 ^a,^*
PMMA SDS	0.227	4.74 ± 0.48 ^a,^*
PMMA SDS/HPβCD	0.125	6.44 ± 0.16 ^a,^**
PLGA	0.154	3.23 ± 0.25 ^b,^*
PLGA SDS	0.166	4.90 ± 0.22 ^b,^**
PLGA SDS/HPβCD	0.139	8.21 ± 0.72 ^b,^***

Statistical analysis was assessed by one-way ANOVA, followed by Tukey’s multiple comparison test: ^a^ was used for the PMMA group, and ^b^ was used for the PLGA group. The data were divided into groups using Tukey’s posttest, and statistically significant differences were observed with * *p* < 0.05, ** *p* < 0.001, and *** *p* < 0.0001.

**Table 3:** Values of the drug loading percentage of nanofiber membranes.

Fibers	Drug Loading Rate (%)
PMMA SDS	40.6 ± 2.0
PMMA SDS/HPβCD	47.5 ± 2.5
PLGA SDS	41.2 ± 2.6
PLGA SDS/HPβCD	43.0 ± 0.3

**Table 4:** Mathematical models and curve fitting parameters for the SDS release profiles.

Mathematical Models	PMMA SDS	PMMA SDS/HPβCD	PLGA SDS	PLGA SDS/HPβCD
First Order	r^2^ = 0.7354	r^2^ = 0.8155	r^2^ = 0.8017	r^2^ = 0.6991
	k = 1.19 × 10^−4^	k = 1.57 × 10^−4^	k = 3.21 × 10^−4^	k = 4.54 × 10^−4^
Higuchi	r^2^ = 0.9087	r^2^ = 0.8489	r^2^ = 0.8900	r^2^ = 0.9376
	k = 0.9949	k = 1.3800	k = 0.4405	k = 0.5440
Peppas	r^2^ = 0.9843	r^2^ = 0.9915	r^2^ = 0.9809	r^2^ = 0.9600
	k = 2.0659	k = 2.1388	k = 2.2780	k = 2.5366
	n = 0.12519	n = 0.1434	n = 0.2284	n = 0.2994
Weibull	r^2^ = 0.9751	r^2^ = 0.9870	r^2^ = 0.9648	r^2^ = 0.9416
	k = 1.1440	k = 1.2107	k = 1.2312	k = 1.2572
	b = 0.1467	b = 0.1688	b = 0.2657	b = 0.3472
Zero Order	r^2^ = 0.6280	r^2^ = 0.7598	r^2^ = 0.7501	r^2^ = 0.7508
	k = 0.0115	k = 0.0281	k = 0.0365	k = 0.0391
Hixon and Crowell	r^2^ = 0.6823	r^2^ = 0.7798	r^2^ = 0.7599	r^2^ = 0.6198
	k = 0.0083	k = 0.01141	k = 0.0213	k = 0.0283

**Table 5:** Inhibition halo (mm) of tested materials.

Groups	*S. aureus*	*E. coli*
Negative Control	0	0
CHX	0.5	0.5
PMMA	0	0
PLGA	0	0
PMMA SDS	0	0
PMMA SDS/HPβCD	0	0
PLGA SDS	0.2	0.3
PLGA SDS/HPβCD	0.7	0.5

## Data Availability

Not applicable.

## Supplementary Materials

The following supporting information can be downloaded at: https://www.mdpi.com/article/10.3390/pharmaceutics15071890/s1, Figure S1: PMMA histograms; Figure S2: PLGA histograms; Figure S3: Contact angles images; Figure S4: Calibration curves; Figure S5: Microscopy images of a pseudo-wound for PMMA systems; Figure S6: Microscopy images of a pseudo-wound for PLGA systems.
