# Supplementary material for: Combining Polymer and Cyclodextrin Strategy for Drug Release of Sulfadiazine from Electrospun Fibers
Source: Pharmaceutics. 2023 Jul 5;15(7):1890. doi: 10.3390/pharmaceutics15071890 (PMC10386385; doi:10.3390/pharmaceutics15071890)
Supplement: Supplementary file 1 [file pharmaceutics-15-01890-s001.zip › pharmaceutics-2465495-supplementary.pdf]

Supplementary Material

Combining Polymer and Cyclodextrin Strategy for Drug Release of Sulfadiazine from Electrospun Fibers

Diego C. Morais [1], Marina L. Fontes [2], Analú B. Oliveira [3], Paulo R. Gabbai-Armelin [3], Túlio M. Ferrisse [4], Luiz F. C. De Oliveira [5], Fernanda Lourenção Brighenti [3], Hernane S. Barud [2] and Frederico B. De Sousa [1,*]

[1] Laboratório de Sistemas Poliméricos e Supramoleculares (LSPS), Instituto de Física e Química, Universidade Federal de Itajubá (UNIFEI), Itajubá 37500-903, MG, Brazil; diegocapronimorais@gmail.com
[2] Laboratório de Biopolímeros e Biomateriais, Universidade de Araraquara (UNIARA), Araraquara 14801-340, SP, Brazil; marinafontes29@gmail.com (M.L.F.); hernane.barud@gmail.com (H.S.B.)
[3] Department of Morphology and Pediatric Dentistry, School of Dentistry, São Paulo State University (UNESP), Araraquara 14801-903, SP, Brazil; analu.oliveira@unesp.br (A.B.O.); paulogabbai@gmail.com (P.R.G.-A.); f.brighenti@unesp.br (F.L.B.)
[4] Department of Dental Materials and Prosthodontics, School of Dentistry, São Paulo State University (Unesp), Araraquara 14801-903, SP, Brazil; tuliomferrisse@gmail.com
[5] Núcleo de Espectroscopia E Estrutura Molecular—Departamento de Química—ICE, Universidade Federal de Juiz de Fora (UFJF), Juiz de Fora 36036-900, MG, Brazil; luiz.oliveira@ufjf.br
* Correspondence: fredbsousa@unifei.edu.br

S1: Histograms

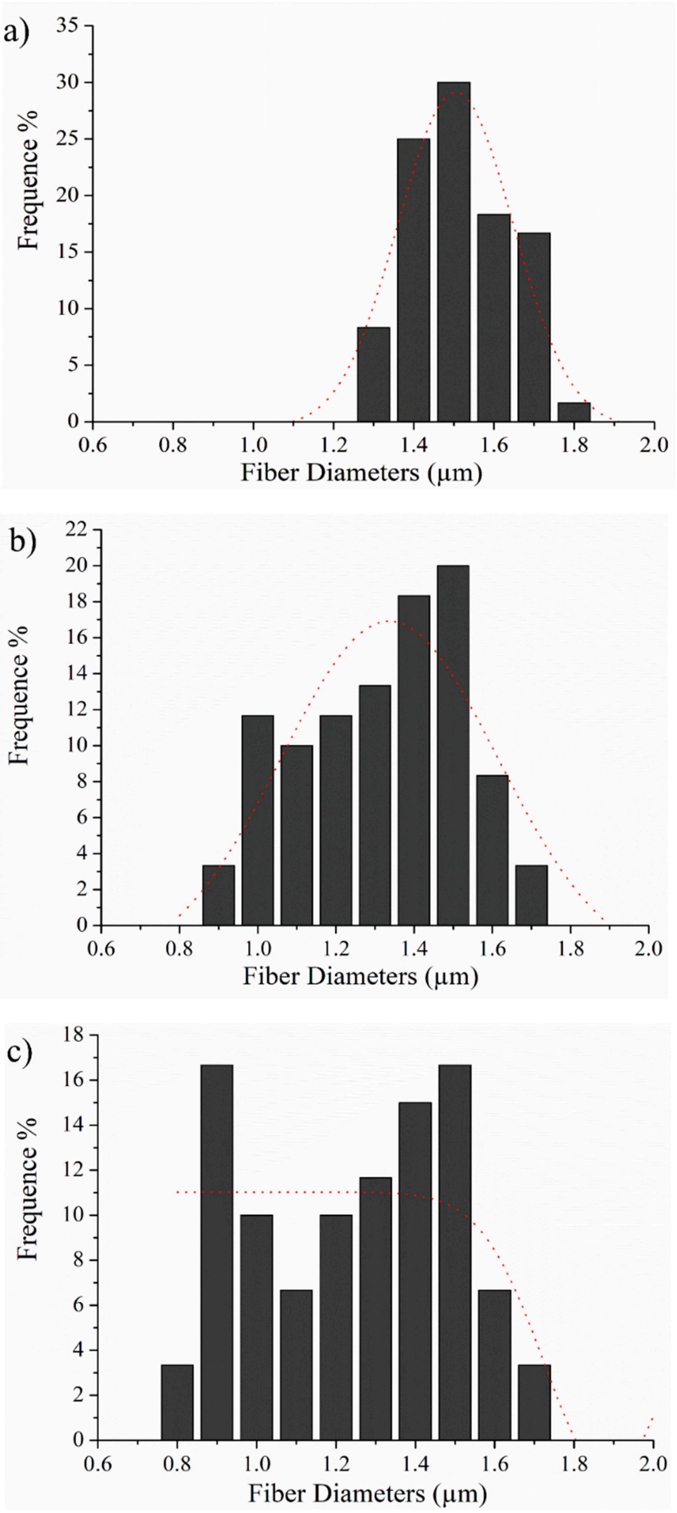

Figure S1: Histograms of fiber diameter distribution for: (a) PMMA, (b) PMMA SDS, and (c) PMMA SDS/HPβCD.

S2: Histograms

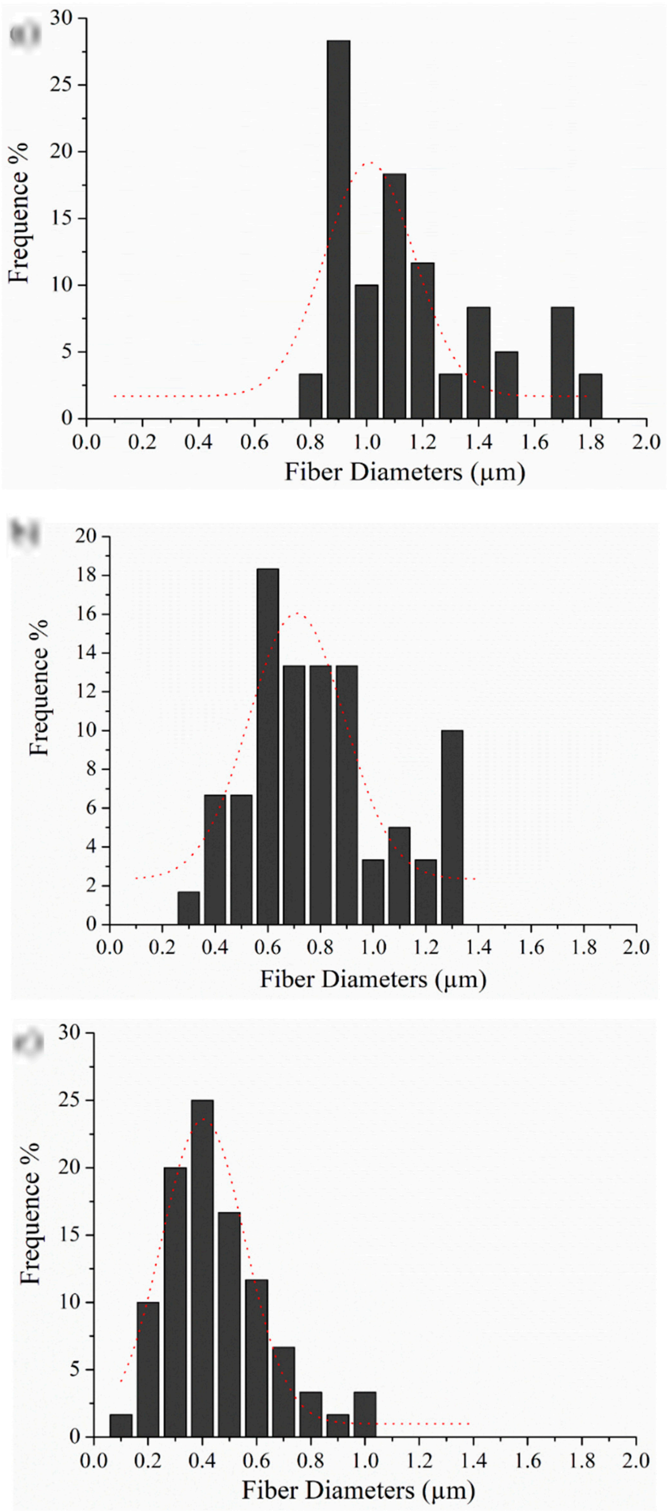

Figure S2: Histograms of fiber diameter distribution for: (a) PLGA, (b) PLGA SDS, and (c) PLGA SDS/HPβCD.

S3: Contact angle

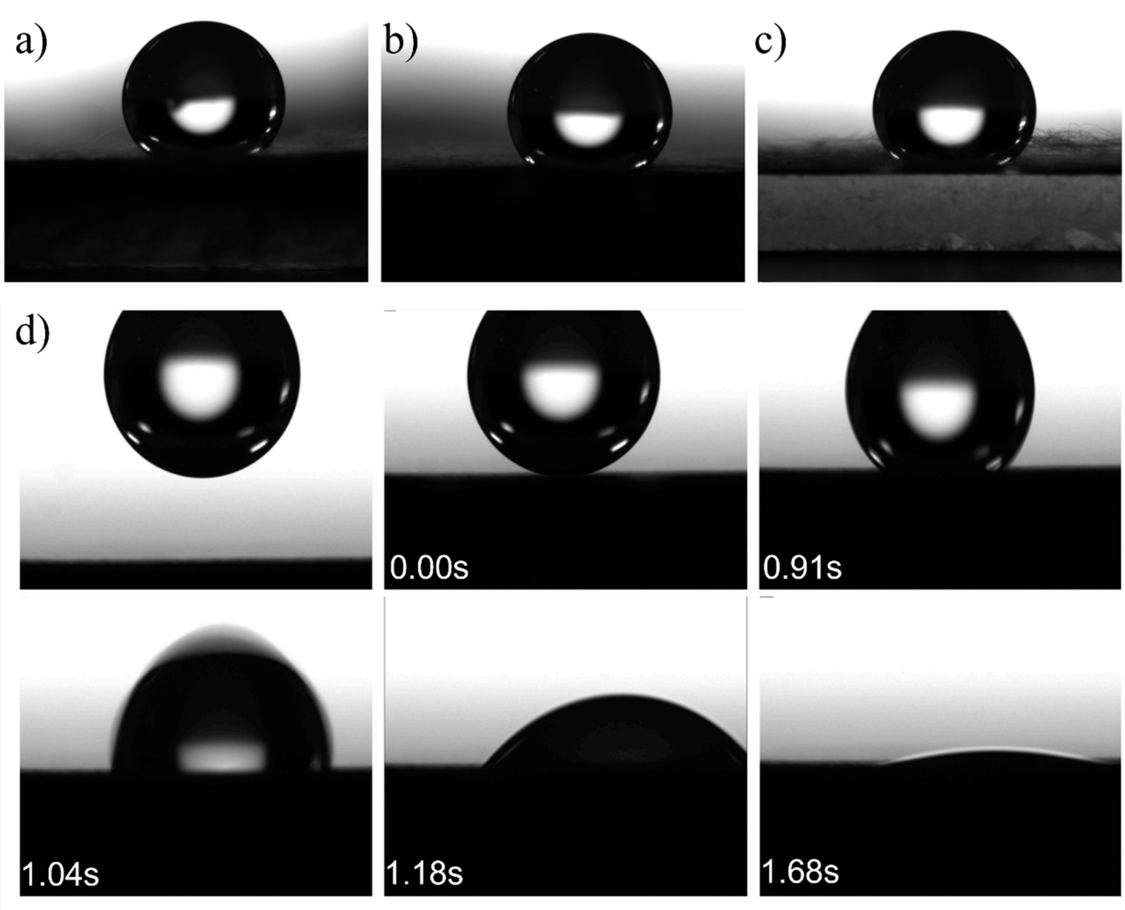

Figure S3: water contact angles images for the electrospun systems: (a) PMMA, (b) PMMA SDS, (c) PMMA SDS/HPβCD; (d) PLGA with the capture of video frames.

S4: calibration curve

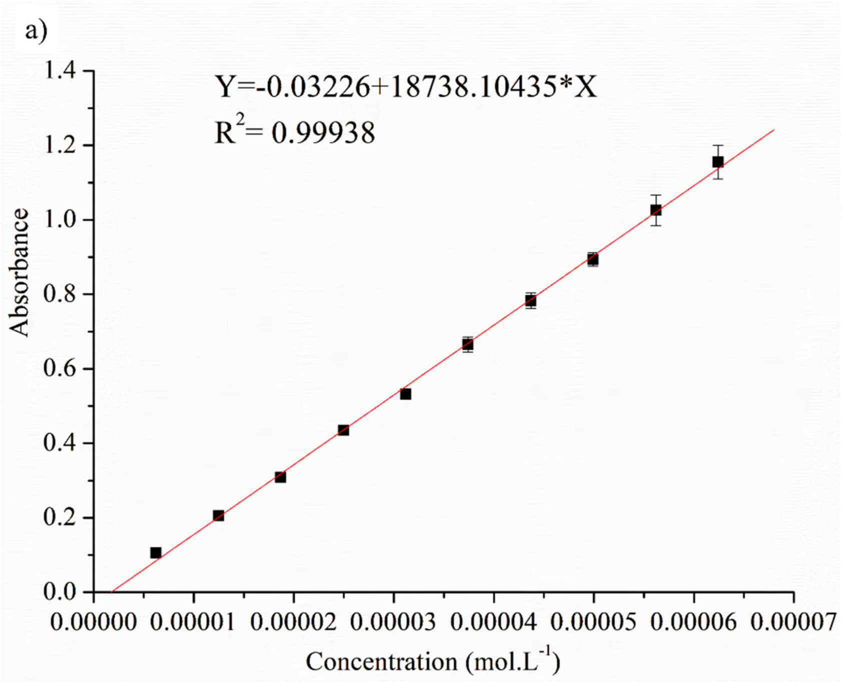

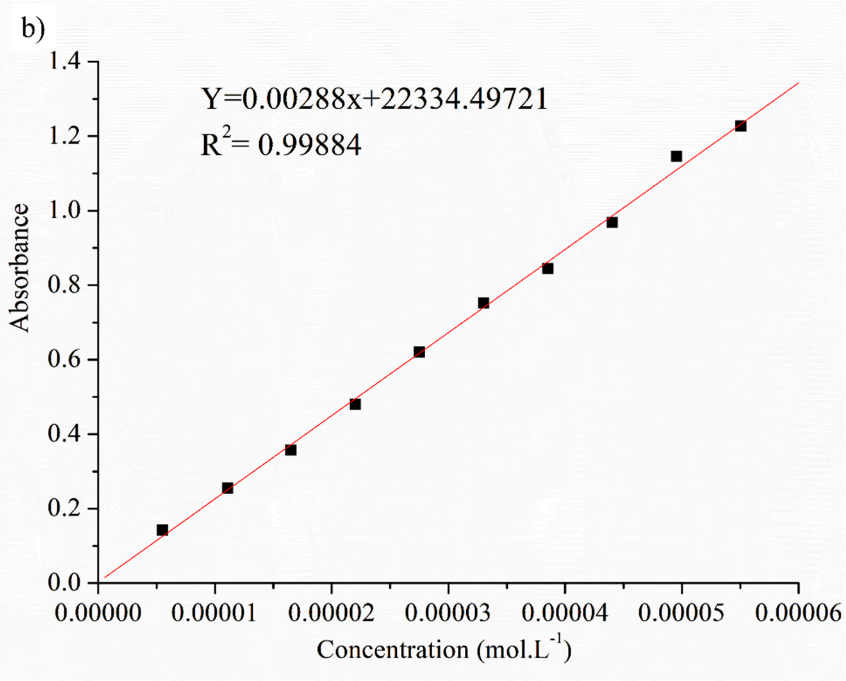

Figure S4: Calibration curve for SDS drug: (a) without HPβCD and (b) with HPβCD.

S5: microscopies images

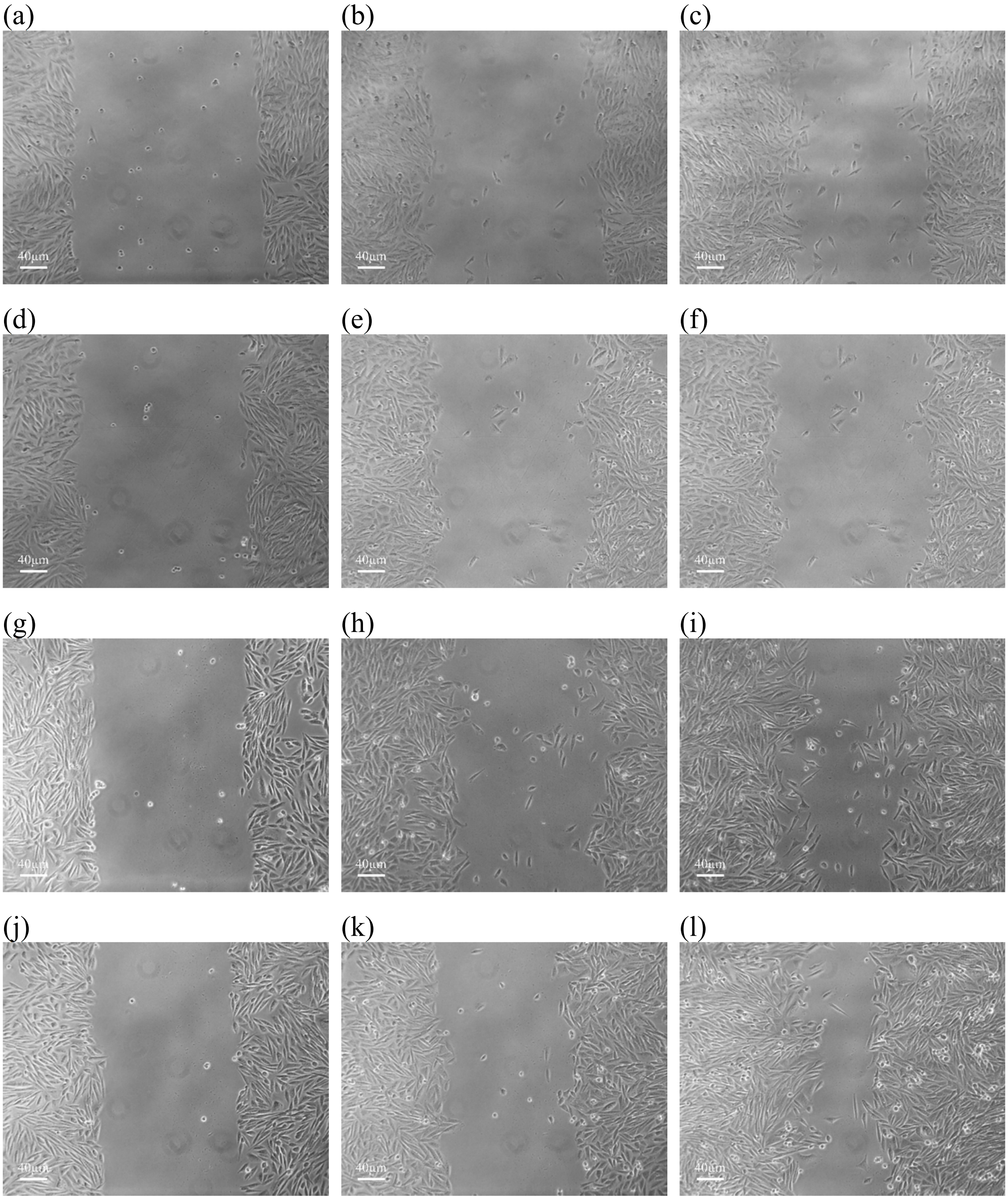

Figure S5: Microscopies images of a pseudo-wound created in a fibroblast cell L929 monolayer, at 0 hour (left column), at 10 hours (middle column), and at 24 hours (right column). The pseudo-wounds were treated with (a–c) PMMA, (d–f) PMMA SDS, (g–i) PMMA SDS/HPβCD, and (j–l) control group.

S6: microscopies images

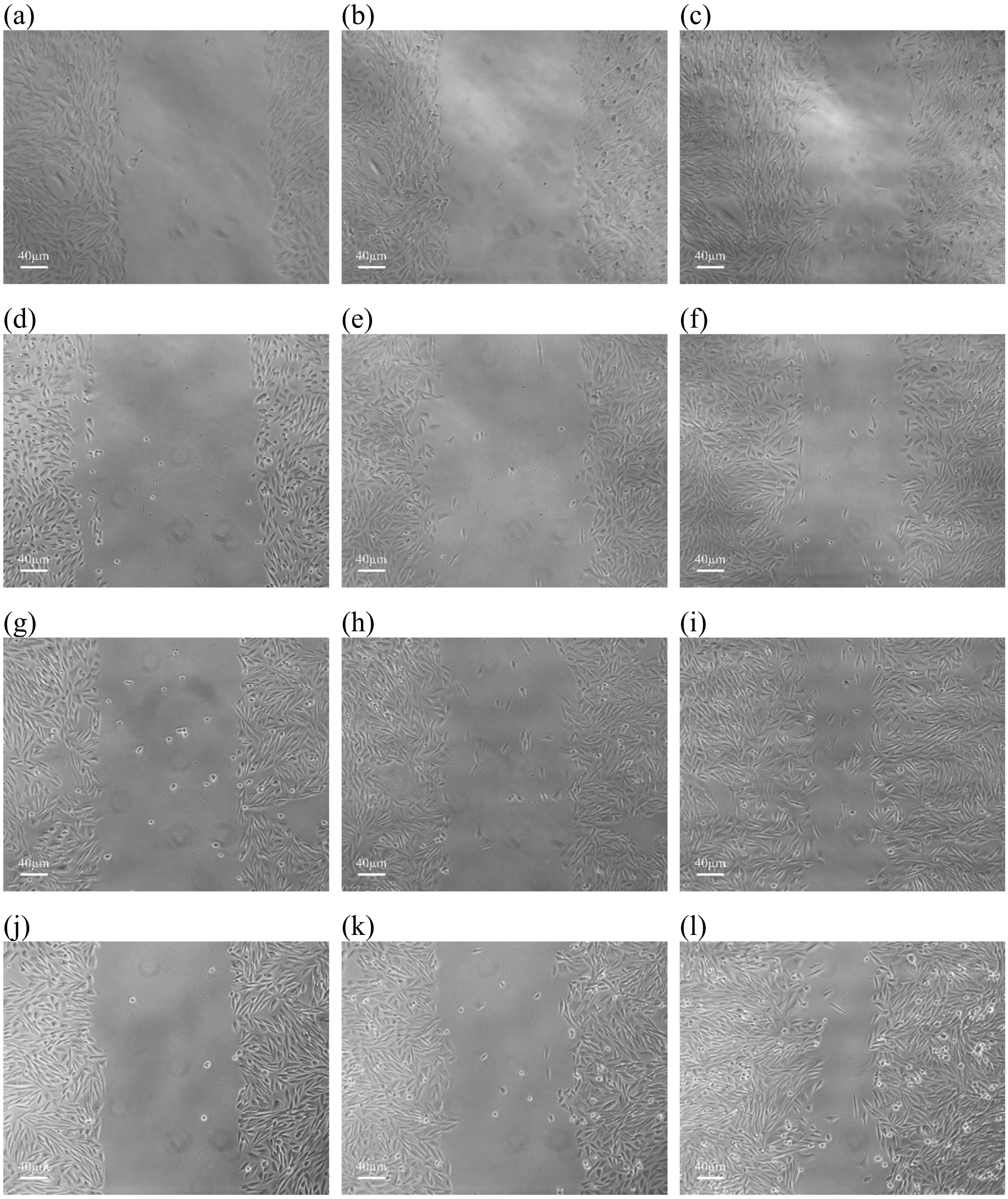

Figure S6: Microscopies images of a pseudo-wound created in a fibroblast cell L929 monolayer, at 0 hour (left column), at 10 hours (middle column), and at 24 hours (right column). The pseudo-wounds were treated with (a–c) PLGA, (d–f) PLGA SDS, (g–i) PLGA SDS/HPβCD, and (j–l) control group.
